# AI-Powered Insights into Drug Resistance in Gastric Cancer: A Path Toward Precision Therapy

**DOI:** 10.5812/ijpr-159954

**Published:** 2025-05-25

**Authors:** Negar Mottaghi-Dastjerdi, Mohammad Soltany-Rezaee-Rad

**Affiliations:** 1 Department of Pharmacognosy and Pharmaceutical Biotechnology, School of Pharmacy, Iran University of Medical Sciences, Tehran, Iran; 2 Behestan Innovation Factory, Behestan Darou, Tehran, Iran

**Keywords:** Artificial Intelligence, Biomarker, Drug resistance, Gastric Cancer, Precision medicine

## Abstract

**Context:**

Gastric cancer (GC) is a major global health burden, with drug resistance representing a critical barrier to effective treatment. Understanding the mechanisms underlying drug resistance and leveraging advanced technologies, such as artificial intelligence (AI), are essential for developing innovative therapeutic strategies.

**Evidence Acquisition:**

This review systematically examines the primary mechanisms of drug resistance in GC, organized into eight categories: Reduced drug uptake, enhanced drug efflux, impaired pro-drug activation or increased inactivation, molecular target alterations, enhanced DNA damage repair, imbalance in apoptotic regulation, tumor microenvironment modifications, and phenotypic changes. Additionally, the role of AI in addressing these challenges is explored, with a focus on omics-driven insights, pathway analysis, biomarker discovery, and modeling drug-response relationships.

**Results:**

The review highlights the transformative potential of AI in advancing precision therapy for GC. Key applications include therapeutic stratification, optimization of drug combinations, adaptive therapy design, and integration with clinical workflows. Challenges such as data quality, model interpretability, and the need for interdisciplinary collaboration are identified, along with strategies to address these barriers. Future directions emphasize the development of explainable AI models, integration of multi-omics and real-time patient data, and AI-driven drug discovery targeting resistance pathways.

**Conclusions:**

By bridging research and clinical practice, AI offers a promising path to more effective, personalized, and adaptive therapeutic strategies for GC. Overcoming existing challenges and leveraging AI's potential can significantly improve treatment outcomes and address the pressing issue of drug resistance in GC.

## 1. Context

### 1.1. Gastric Cancer: A Precision Medicine Challenge

Gastric cancer (GC) remains a significant global health challenge, ranking among the leading causes of cancer-related mortality worldwide. In 2020, it was estimated that there were approximately 1.1 million new cases and 770,000 deaths attributed to GC, with incidence rates being on average twice as high in males as in females (1). The burden of this disease is particularly pronounced in Eastern Asia, where countries such as Japan, Mongolia, and the Republic of Korea report the highest incidence rates globally (1).

A critical challenge in the management of GC is the considerable variability in patient responses to therapies. This heterogeneity is influenced by factors such as genetic mutations, epigenetic alterations, and the tumor microenvironment, all of which contribute to differing therapeutic outcomes. For instance, variability in immune response and tumor biology across individuals means that immunotherapy does not uniformly benefit all patients with GC (2). Additionally, the cellular and molecular heterogeneity of GC leads to variable results in chemotherapy, complicating treatment strategies (3).

This variability underscores the necessity for precision medicine approaches tailored to the unique molecular profiles of individual patients, aiming to enhance therapeutic efficacy and improve survival outcomes.

### 1.2. Drug Resistance: A Barrier to Effective Treatment

Drug resistance is a major barrier to effective GC treatment, driven by genetic, epigenetic, and microenvironmental factors that contribute to therapy failure and disease progression.

#### 1.2.1. Genetic Modifications

Genomic instability, copy number alterations, and genetic polymorphisms are among the critical factors influencing drug response and resistance development (4). Alterations in oncogenes and tumor suppressor genes can drive resistance by activating survival pathways or impairing apoptotic mechanisms. For instance, mutations in genes such as TP53, HER2, and MET have been associated with resistance to targeted therapies in GC (5, 6).

#### 1.2.2. Epigenetic Modifications

Epigenetic changes — such as DNA methylation, histone modification, chromatin remodeling, and noncoding RNAs — play a major role in cancer drug resistance. Altered gene expression, epithelial-mesenchymal transition (EMT), and cancer stem cell maintenance driven by elements like miR-21, HOTAIR, and SWI/SNF complexes contribute to resistance. Targeted therapies, including DNA methyltransferase (DNMT) and histone deacetylase (HDAC) inhibitors, show potential in reversing this effect. Epigenetic biomarkers are also emerging as tools for personalized treatment and overcoming resistance (4).

#### 1.2.3. Tumor Microenvironment

The tumor microenvironment (TME) contributes to treatment resistance through interactions with immune-suppressive cells such as cancer-associated fibroblasts (CAFs), myeloid-derived suppressor cells (MDSCs), and tumor-associated macrophages (TAMs). Metabolic reprogramming, hypoxia, and extracellular matrix (ECM) remodeling hinder immunity and drug delivery, while the microbiota affects metabolism and therapy response. Targeting these components offers promising strategies to overcome resistance and improve outcomes (4).

Overcoming resistance in GC requires precision medicine tailored to each patient’s molecular profile. Integrating genomic, epigenomic, and microenvironmental data enables the identification of targeted therapies, offering improved treatment outcomes.

### 1.3. Role of Artificial Intelligence in Precision Oncology

Artificial intelligence is transforming precision oncology by tackling drug resistance and enabling personalized therapy. It integrates multi-omics data to uncover resistance mechanisms and identify biomarkers, guiding targeted treatments tailored to each patient (7). Moreover, AI models predict individual treatment responses, helping clinicians choose the most effective strategies. For example, AI has been used to forecast drug responses at single-cell resolution, enhancing outcomes (8).

This review bridges AI insights with clinical applications in precision oncology, highlighting current methods for addressing drug resistance. It aims to support the integration of AI into practice to advance personalized treatments and improve patient outcomes.

## 2. Drug Resistance Mechanisms in Gastric Cancer

Bioinformatics and systems biology have transformed the discovery of genes and pathways driving cancer progression and drug resistance. Integrating multi-omics data has revealed key dysregulated pathways and hub genes linked to GC resistance. Network and enrichment analyses provide insights into biomarkers and therapeutic targets, supporting precision oncology strategies to overcome resistance and enhance outcomes (9-15).

**Figure 1.**
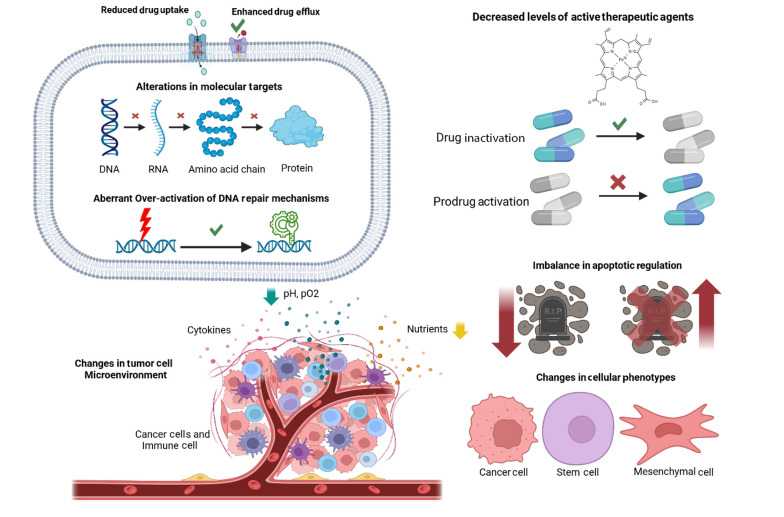
Drug resistance mechanisms in gastric cancer (GC) (https://BioRender.com/d69h482).

The main mechanisms of drug resistance in GC fall into eight categories (Figure 1): Reduced drug uptake, increased drug efflux, impaired activation or enhanced inactivation of drugs, molecular target changes, enhanced DNA repair, dysregulated apoptosis, tumor microenvironment alterations, and phenotypic shifts such as EMT and stem-like traits (16).

### 2.1. Reduced Drug Uptake

Reduced drug uptake is a key mechanism of chemoresistance in GC, driven by altered expression or genetic variants of transport proteins. The down-regulation of CTR1 (SLC31A1 gene) reduces cisplatin sensitivity, while higher expression of SLCO1B3 and other organic anion-transporting polypeptides (OATPs) isoforms (2B1, 3A1, 4A1, 5A1) facilitates the uptake of irinotecan, docetaxel, and methotrexate. The cancer-specific variant of OATP1B3 is highly expressed in GC, though its functional role remains unclear. Additionally, ENT1 contributes to pyrimidine analog uptake, but 5-fluorouracil (5-FU) resistance in GC suggests alternative mechanisms beyond ENT-mediated uptake. These transporters collectively influence drug sensitivity in GC (16-18).

### 2.2. Enhanced Drug Efflux

Genetic alterations in ATP-binding cassette (ABC) transporters contribute significantly to multidrug resistance (MDR) in GC. MDR1 (ABCB1) expression is regulated by deoxycytidine triphosphate pyrophosphatase 1 (DCTPP1), which influences promoter methylation; low DCTPP1 levels increase promoter methylation and reduce MDR1 expression, affecting resistance to cisplatin, oxaliplatin, and epirubicin. MRP1 (ABCC1) overexpression, often observed in cisplatin-resistant GC, is linked to acquired resistance. MRP2 (ABCC2) expression is influenced by single nucleotide polymorphisms (SNPs), such as the c.-24C > T variant, which affects drug response to oxaliplatin. The MRP4 (ABCC4) is highly expressed in cisplatin-resistant GC, with genetic inhibition improving sensitivity. BCRP (ABCG2) expression is associated with poor survival and resistance to cisplatin and 5-fluorouracil (5-FU), while genetic manipulation strategies like ribozymes have shown potential to overcome resistance. Similarly, copper transporters ATP7A and ATP7B exhibit increased expression in oxaliplatin- and cisplatin-resistant GC, though their precise genetic regulation requires further investigation. These genetic changes underscore potential targets for combating GC chemoresistance (16, 17, 19, 20).

### 2.3. Decreased Levels of Active Therapeutic Agents

Chemoresistance in GC is driven by changes in metabolic enzymes that inactivate antitumor drugs or reduce pro-drug activation, resulting in lower intracellular levels of active agents. CYP2A6, responsible for converting tegafur to 5-fluorouracil (5-FU), is affected by single nucleotide polymorphisms (SNPs) that reduce its activity, impacting tumor sensitivity to 5-FU. Thymidine phosphorylase (TP), crucial for 5-FU activation, shows variable correlations with 5-FU sensitivity and patient prognosis. Overexpression of carboxylesterase 2 (CES2) enhances the activation of capecitabine and irinotecan, while dihydropyrimidine dehydrogenase (DPD) overexpression reduces sensitivity to 5-FU. Metallothioneins (MTs) are linked to resistance to irinotecan and cisplatin, with conflicting data on their role in prognosis. Glutathione-S-transferase (GST) enzymes, particularly GSTP1, contribute to resistance by forming inactive drug conjugates with glutathione, although specific variants like GSTP1*B are associated with better responses to 5-FU and oxaliplatin. UDP-glucuronosyltransferases (UGTs), including UGT1A1, influence irinotecan metabolism, with polymorphisms linked to variable outcomes in GC treatment. These genetic and expression changes highlight key mechanisms driving chemoresistance in GC (16, 21-23).

### 2.4. Alterations in Molecular Targets

Chemoresistance in GC is influenced by genetic changes in molecular targets critical for drug efficacy. Thymidylate synthase (TS), inhibited by 5-fluorouracil (5-FU), exhibits polymorphisms such as 2R/2R and 2R/3R genotypes in the TS enhancer region, which correlate with shorter overall survival (OS) in patients treated with 5-FU-based chemotherapy. Variations in DNA topoisomerase II (TOPO II) expression are linked to resistance against doxorubicin, while high β-tubulin-III (TUBB3) expression, a target of taxanes, is associated with resistance to docetaxel and paclitaxel. Additionally, microtubule-associated protein tau (MAPT) inversely correlates with paclitaxel sensitivity.

Tyrosine kinase receptors also play a role, with epidermal growth factor receptor (EGFR) overexpression common in GC but showing limited clinical efficacy with anti-EGFR therapies like cetuximab. HER2, targeted by trastuzumab, shows improved OS in HER2-positive patients, but resistance correlates with lower HER2 copy numbers. Vascular endothelial growth factor receptor-2 (VEGFR-2), targeted by ramucirumab and apatinib, influences survival outcomes, with apatinib demonstrating effectiveness by reversing MDR1 and BCRP-mediated resistance. For vascular endothelial growth factor (VEGF), low expression correlates with poor outcomes in bevacizumab-treated patients, while variants in the VEGF pathway (e.g., VEGF-A and VEGF-C) may predict treatment response. These genetic and molecular changes underscore the complexity of chemoresistance mechanisms in GC (16, 24-27).

### 2.5. Aberrant Over-Activation of DNA Repair Mechanisms

Chemoresistance in GC is heavily influenced by genetic changes in DNA repair mechanisms, which enhance tumor cell survival by counteracting drug-induced damage. Overexpression of ERCC1, a key player in the nucleotide-excision repair (NER) system, is linked to poor outcomes in GC patients treated with platinum-based therapies, with polymorphisms such as rs11615 and rs3212986 further affecting chemotherapy sensitivity. MicroRNAs such as miR-122 and miR-139-5p inversely regulate ERCC1 expression, with their induction restoring cisplatin sensitivity. Similarly, ERCC2 and ERCC4 overexpression contribute to resistance, though limited clinical evidence exists for ERCC4.

In base-excision repair (BER), increased XRCC1 expression is associated with cisplatin resistance, while its rs25487 polymorphism correlates with poor outcomes in oxaliplatin-treated patients. Mismatch repair (MMR) deficiencies, including loss of MLH1, result in microsatellite instability (MSI), present in up to 30% of GC cases, and contribute to reduced sensitivity to 5-fluorouracil (5-FU)-based treatments. Low MSI status, however, is linked to improved disease-free survival with adjuvant chemotherapy.

Finally, in homologous recombination (HR), reduced BRCA1 and BRCA2 expression is observed in GC, though the BRCA1 rs799917 polymorphism positively impacts survival in patients treated with taxane and cisplatin therapies. These genetic alterations underscore the role of DNA repair systems in mediating chemoresistance in GC (16, 28-33).

### 2.6. Imbalance in Apoptotic Regulation

Chemoresistance in GC is strongly influenced by genetic alterations in pro-apoptotic factors, impairing drug-induced apoptosis. TP53 mutations, including loss-of-function and gain-of-function variants such as those at Arg175, Gly245, and Arg248, result in diminished or oncogenic p53 activity, leading to enhanced HER2 expression and poorer outcomes with drugs like 5-fluorouracil (5-FU), paclitaxel, and cisplatin. The rs1042522 variant of TP53 is linked to worse response rates to chemotherapy. Loss of CDKN2A expression, due to promoter hypermethylation, is common in Epstein-Barr virus-associated GC and correlates with reduced efficacy of 5-FU therapy. Low expression of intrinsic pathway proteins, including BAX, BAK, BAD, BIM, and BLID, further reduces sensitivity to treatments like docetaxel, cisplatin, and 5-FU. Dysregulation of BIM and BAD by microRNAs, such as miR-BART20-5p and miR-501, promotes chemoresistance, while down-regulation of BLID inactivates caspases 3 and 9, fostering doxorubicin resistance. Impairments in the extrinsic apoptosis pathway, such as FADD loss and miR-633 overexpression targeting FADD, also contribute to resistance. These genetic changes highlight key apoptotic mechanisms driving GC chemoresistance (16, 34-37).

Chemoresistance in GC is also driven by genetic and molecular changes in survival pathways, which inhibit apoptosis and promote tumor cell resistance. Hyperactivation of the NF-κB pathway, commonly observed in GC, up-regulates anti-apoptotic factors like survivin, BCL-XL, and XIAP, contributing to cisplatin resistance and acquired chemoresistance. Elevated survivin serum levels correlate with responses to chemotherapy in advanced GC. The Wnt/β-catenin pathway, frequently hyperactive in GC, is exacerbated by Helicobacter pylori infection, which induces SOX9, NANOG, and OCT4 expression. Mutations in CTNNB1, APC, and FBXW7 are linked to poor overall survival (OS) and progression-free survival (PFS) in chemotherapy-treated patients. Loss of E-cadherin, which increases β-catenin levels, is more prevalent in chemoresistant GC. Hyperactivation of the Hedgehog pathway, influenced by *H. pylori* and chemotherapy exposure, up-regulates GLI1, GLI2, and SHH, contributing to resistance to 5-FU and doxorubicin. The Notch pathway, up-regulated in cisplatin-resistant GC, promotes MDR1 and MRP1 expression via lncRNA AK022798, reducing caspase-3 and caspase-8 activity. High Notch1 expression correlates with non-responsiveness to 5-FU and cisplatin. Aberrations in the Hippo pathway lead to nuclear accumulation of YAP1 and TAZ, linked to poor responses to adjuvant chemotherapy. Increased activity of PI3K/AKT and JAK/STAT3 pathways is associated with reduced sensitivity to cisplatin and trastuzumab. These molecular changes collectively enhance GC survival and chemoresistance (16, 38-44).

### 2.7. Changes in Tumor Cell Microenvironment

Chemoresistance in GC is influenced by genetic and molecular changes within the tumor microenvironment, including interactions with stromal cells, blood vessels, and inflammatory cells. Hypoxia-inducible factor 1-alpha (HIF-1α), up-regulated in hypoxic conditions, promotes resistance to platinum derivatives by altering the expression of miR-27a, miR-421, and long noncoding RNA (lncRNA) PVT1, while also increasing MDR1, MRP1, and BCL-2 levels. Stanniocalcin 1 (STC1), enhanced under hypoxia, contributes to cisplatin resistance by up-regulating BCL-2 and reducing caspase activity.

Inflammation-driven factors also play a role. NR4A2, induced by prostaglandin E2, inhibits apoptosis and correlates with poor survival in 5-fluorouracil (5-FU)-treated patients. Cytokines like interleukin (IL)-6, IL-8, and IL-11 promote resistance through pathways involving NF-κB, ABCB1, and BCL-2, while IL-33 activates the JNK pathway to prevent apoptosis. Autocrine signaling via CCL2 maintains cisplatin resistance by inactivating autophagy through PI3K-AKT-mTOR signaling.

In exploring the molecular mechanisms underlying chemoresistance, the role of autophagy-related genes, such as FOXO3 and GAPDH, has been highlighted in various cancers, underscoring the potential for targeting autophagic pathways to enhance treatment efficacy. Overexpression of miR-23b-3p reverses resistance mediated by ATG-12 and HMGB2, while ATG-5 up-regulation is linked to shorter overall survival. Exosomes further contribute to chemoresistance. Mesenchymal stem cell-derived exosomes up-regulate ABC pumps, and tumor-associated macrophage-derived exosomes transfer miR-21a-5p, enhancing cisplatin resistance via the PI3K/AKT pathway.

Additionally, metabolic adaptations, such as glycolysis up-regulation and fatty acid oxidation driven by lncRNA HCP5, support resistance to 5-FU and oxaliplatin. These molecular and genetic changes highlight the complex role of the tumor microenvironment in GC chemoresistance (15, 45-52).

### 2.8. Changes in Cellular Phenotypes

Chemoresistance in GC arises from genetic changes and phenotypic shifts associated with EMT and cancer stem cells (CSCs). EMT, driven by factors such as transforming growth factor-beta (TGF-β), hepatocyte growth factor (HGF), and hypoxia-inducible factor 1-alpha (HIF-1α), promotes resistance through the up-regulation of CD168 (HMMR), vimentin, and N-cadherin. MicroRNA regulation further contributes, with miR-577 enhancing TGF-β signaling and increasing oxaliplatin resistance, and miR-187 up-regulating ERCC1/4, reducing cisplatin sensitivity.

Cancer stem cells, marked by surface proteins like CD44, CD24, CD133, CXCR4, and EpCAM, display intrinsic chemoresistance. CD44 splicing variants activate pathways such as Hedgehog, VEGF, and c-Met, driving resistance to 5-fluorouracil (5-FU), cisplatin, and anthracyclines. High CD133 expression up-regulates MDR1 and BCL-2 via the PI3K/AKT pathway, correlating with shorter overall survival (OS) in cisplatin/5-FU-treated patients. Additionally, CXCR4 expression in diffuse-type GC is linked to docetaxel resistance, while aldehyde dehydrogenase 1 (ALDH1) isoenzymes protect CSCs from oxidative damage, reducing 5-FU efficacy.

Key EMT regulators, such as LGR5 and DCLK1, promote resistance by activating pathways like Wnt/β-catenin and Notch, which enhance stemness and EMT markers, including SOX2, OCT4, and SNAIL. Prolonged exposure to oxaliplatin, doxorubicin, or trastuzumab induces EMT through β-catenin, Fas, and TGF-β-miR-200c-ZEB2 signaling. Resistance is further supported by YAP1-PI3K activation in trastuzumab-treated cells.

Lastly, CSCs characterized by low CD71 expression or high side-population cell activity, enriched during 5-FU treatment, exhibit elevated BCRP and MDR1 levels. These changes, along with SOX2-mediated ABCG2 expression, confer resistance to 5-FU, cisplatin, and doxorubicin, underscoring the role of EMT and CSCs in GC chemoresistance (16, 42, 53-63).

## 3. Artificial Intelligence Applications in Understanding and Predicting Drug Resistance in Gastric Cancer

**Figure 2.**
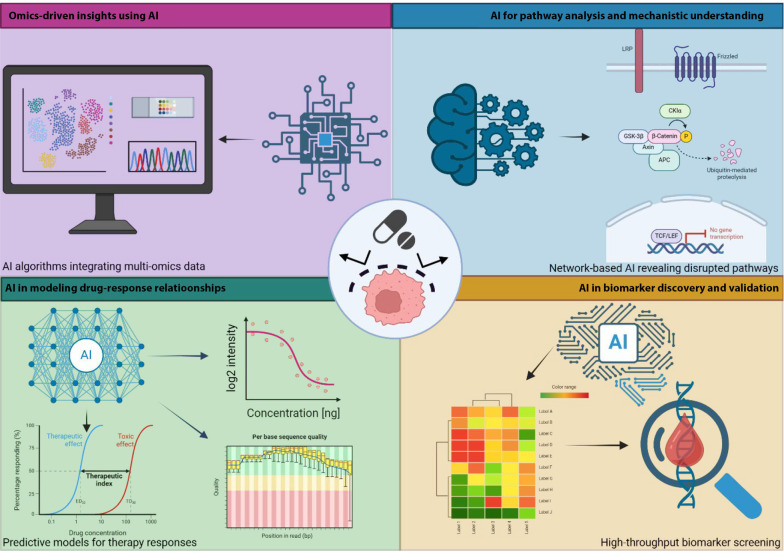
Artificial intelligence (AI) applications in understanding and predicting drug resistance in gastric cancer (GC): AI integrates multi-omics data, uncovers resistance pathways, identifies predictive biomarkers, and models drug sensitivity to optimize therapies in GC (https://BioRender.com/q58w575).

Artificial intelligence has transformed GC drug resistance research by integrating multi-omics data, revealing disrupted pathways, and identifying predictive biomarkers. Machine learning (ML) models help uncover resistance mechanisms, forecast treatment outcomes, and guide personalized therapies, offering new solutions to clinical challenges (Figure 2). 

### 3.1. Omics-Driven Insights Using Artificial Intelligence

The AI has significantly transformed omics analysis in GC by integrating genomic, transcriptomic, and proteomic data to uncover resistance mechanisms and biomarkers. Machine learning (ML) and deep learning (DL) algorithms efficiently analyze large datasets to reveal complex gene and protein interactions (64-68). Convolutional neural networks (CNNs) and support vector machines (SVMs) have been applied to multi-omics data to identify genetic mutations, transcriptomic signatures, and proteomic alterations associated with drug resistance (66, 67, 69, 70). The AI models analyzing transcriptomic and proteomic data have identified pathways such as PI3K/AKT and Wnt/β-catenin, and proteins like BCL-2, MDR1, and β-catenin, as drivers of cisplatin and 5-fluorouracil (5-FU) resistance in GC. Genes such as COL1A1, THBS2, and SPP1 have also been linked to prognosis and programmed death-ligand 1 (PD-L1) expression, suggesting roles in immunotherapy response (71, 72). Recent studies demonstrate that AI can effectively identify drug resistance biomarkers in GC. For instance, one study utilized AI to analyze tumor genetics and predict treatment response by detecting mutations linked to replication stress resistance (73). Another study employed AI on multi-omics data to reveal how inflammatory processes contribute to GC onset, offering insights into potential therapeutic targets (74). These advances underscore AI’s capability to integrate multi-omics data, aiding in decoding GC drug resistance and supporting personalized treatment strategies.

### 3.2. Artificial Intelligence for Pathway Analysis and Mechanistic Understanding

The AI has significantly enhanced our understanding of GC drug resistance by analyzing complex biological networks. Deep learning (DL) models are instrumental in identifying disrupted signaling pathways associated with chemoresistance. For instance, an AI-based network biology study identified key GC resistance-related genes and pathways, such as CTNNB1, BCL2, and TP53, as potential therapeutic targets (75). Furthermore, AI-based tools have been developed to predict treatment responses in GC by analyzing digital pathology images. The DeepRisk network, an AI model, was specifically designed to assess the tumor microenvironment and predict chemotherapy benefits for GC patients, thereby aiding in personalized treatment strategies (76). The integration of AI with multi-omics data has facilitated the identification of molecular subtypes and disrupted pathways in GC, providing insights into resistance mechanisms and guiding the development of targeted therapies (77). These advancements underscore AI’s pivotal role in uncovering complex signaling networks in GC drug resistance, supporting the development of targeted therapies.

### 3.3. Artificial Intelligence in Biomarker Discovery and Validation

The AI has significantly advanced the discovery of biomarkers linked to drug resistance in GC, enabling more personalized treatment through complex data analysis. The AI-driven analyses have identified specific microRNA (miRNA) profiles associated with chemotherapy resistance in GC, underscoring their potential as predictive biomarkers (78). Additionally, AI has been employed to assess the tumor-immune microenvironment in advanced GC, utilizing a digital scoring system to predict immunotherapy benefits and reveal immune-related biomarkers linked to resistance (79). These advancements highlight AI’s crucial role in high-throughput biomarker screening, facilitating the discovery of molecular signatures associated with therapy resistance in GC and guiding personalized treatment strategies.

### 3.4. Artificial Intelligence in Modeling Drug-Response Relationships

The AI has significantly advanced drug-response modeling in GC by utilizing machine learning (ML) to predict sensitivity and resistance to treatments such as cisplatin, 5-fluorouracil (5-FU), and targeted therapies. ML models leveraging genomic and transcriptomic data have demonstrated promise in predicting chemotherapy responses. For instance, one study developed a model incorporating 123 omics features from GC biopsies, achieving an accuracy of 70–80%, thereby highlighting AI’s role in personalizing treatment (80). The integration of radiological and pathological data with AI has also shown promise in predicting chemotherapy response. A recent study combined computed tomography (CT) and whole-slide imaging (WSI) to construct an ML model that effectively predicted pathological responses in GC patients, demonstrating strong area under the curve (AUC) performance and clinical utility (81). These advances illustrate how AI enhances the understanding of drug responses in GC, supporting more effective, personalized treatments.

## 4. Artificial Intelligence-Driven Approaches to Precision Therapy

**Figure 3.**
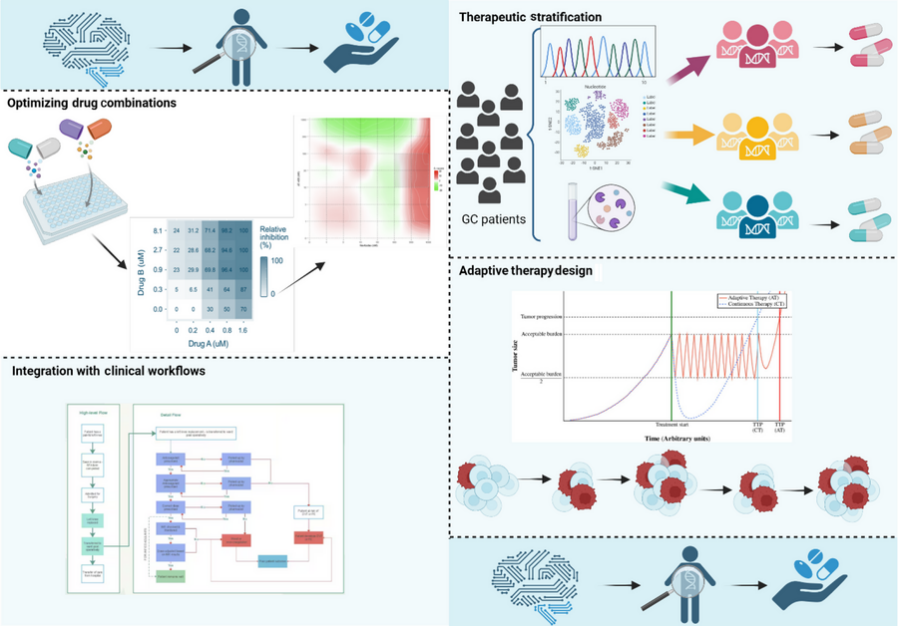
Artificial intelligence (AI)-driven approaches to precision therapy in gastric cancer (GC) (https://BioRender.com/uiwfgcj).

The AI is transforming GC treatment by enabling personalized therapy. This section explores AI applications in patient stratification, drug combinations, adaptive therapies, and clinical integration (Figure 3). 

### 4.1. Therapeutic Stratification

The AI algorithms analyze complex genomic, histopathological, and clinical data to match GC patients with optimal therapies. Deep learning models, for example, assess HER2 status to identify candidates for HER2-targeted treatments like trastuzumab. One study introduced a Multi-Scale Hybrid Vision Transformer model that accurately classifies GC pathology, aiding in treatment decisions. Additionally, AI-driven analysis of digital pathology images has shown promise in identifying candidates for HER2 inhibitors, enhancing personalized treatment strategies (82, 83).

### 4.2. Optimizing Drug Combinations

The AI has emerged as a pivotal tool in optimizing drug combinations to combat resistance in GC. Machine learning (ML) models are increasingly employed to predict synergistic interactions between chemotherapeutic agents and targeted therapies, thereby enhancing treatment efficacy. For instance, deep learning (DL) frameworks have been developed to predict anticancer synergistic drug combinations by integrating chemical descriptors of drugs and genomic data of specific cancer cell lines. These models facilitate the identification of effective drug pairs tailored to individual tumor profiles, potentially improving therapeutic outcomes in GC (84, 85). Additionally, AI-driven platforms such as GraphSynergy have been designed to predict synergistic cancer drug combinations accurately. By analyzing large datasets encompassing drug properties and cancer cell line responses, these platforms can identify promising drug combinations that may overcome resistance mechanisms in GC (86). While these AI methodologies have shown promise in various cancer types, their application specifically to GC is an evolving field. Ongoing research aims to refine these models to account for the unique molecular characteristics of GC, with the goal of developing personalized and effective combination therapies to overcome drug resistance.

### 4.3. Adaptive Therapy Design

Real-time AI systems have been developed to enable dynamic adjustment of treatment strategies based on patient responses in GC. By continuously analyzing patient data, including tumor biomarkers and clinical parameters, AI can recommend modifications to therapy regimens to address emerging resistance or adverse effects. For instance, a study by Zhou et al. introduced an incomplete multimodal data integration framework for GC (iMD4GC) that addresses challenges posed by incomplete multimodal data, enabling precise response prediction and survival analysis. This approach allows for personalized adjustments in treatment strategies based on individual patient data, aiming to maintain treatment efficacy and improve patient quality of life (87). Additionally, AI has been utilized to develop personalized cancer treatment strategies that dynamically adjust treatment to suppress the growth of treatment-resistant populations. For example, Moffitt Cancer Center has employed AI to create adaptive therapy strategies that adjust treatment plans in real-time, enhancing the effectiveness of cancer treatments (88). These advancements highlight the potential of AI in facilitating real-time, adaptive treatment strategies in GC, aiming to enhance therapeutic efficacy and patient outcomes.

### 4.4. Integration with Clinical Workflows

The AI is increasingly being integrated into clinical workflows to enhance decision-making in GC management. AI-driven decision-support tools analyze patient-specific data to generate evidence-based recommendations, facilitating personalized treatment planning. For instance, the development of tools like GastricAITool aims to assist clinicians in diagnosing and prognosticating GC, thereby supporting critical decision-making and enabling personalized strategies (89). Additionally, AI chatbots have been evaluated for their effectiveness in improving patient outcomes, alleviating anxiety, and promoting informed decision-making among GC patients. A comparative study assessed the performance of AI chatbots, including Sider Fusion AI Bot and Perplexity AI, in providing support and information to young adults diagnosed with GC, highlighting their potential to enhance patient engagement and outcomes (90). The integration of AI into clinical workflows aims to enhance decision-making efficiency and improve patient outcomes in GC management. By providing clinicians with advanced tools for early diagnosis, prediction of adverse outcomes, and personalized treatment recommendations, AI has the potential to significantly improve the quality of care for GC patients.

## 5. Challenges and Opportunities of Artificial Intelligence

There are a number of challenges and opportunities related to the application of AI. Table 1 summarizes these points, and in the following section, each challenge and opportunity is discussed in detail.

**Table 1.** Challenges of Artificial Intelligence in Gastric Cancer

Barrier/Challenge	Description
**Barriers to AI implementation**	
Data scarcity	Limited availability of high-quality datasets for model training and validation in GC research.
Dataset biases	Non-representative patient populations or inconsistent data collection methods lead to biased models that may perform poorly in real-world clinical settings.
Standardization issues	Lack of standardized data collection methods affects the reliability and applicability of AI models.
Black box models	Many DL models lack interpretability, making it difficult for clinicians to understand the rationale behind predictions.
Clinical trust and safety	Without transparent reasoning, physicians may be reluctant to trust AI recommendations, impacting patient safety and acceptance in clinical practice.
Need for explainability	Developing interpretable AI models or incorporating explainability features is critical for integration into GC treatment protocols.
Integration challenges	Ensuring that technological advancements in AI translate into practical and effective clinical applications.
**Bridging research and clinical practice**	
Physician trust	Many clinicians are hesitant to rely on AI-driven predictions without clear interpretability and validation in diverse patient populations.
Regulatory compliance	AI-driven decision-support tools must adhere to stringent guidelines set by regulatory agencies such as the FDA and EMA.
Implementation costs	Integrating AI into hospital infrastructures requires substantial investment in technology, training, and data management systems.
Education and training	Ongoing education and training programs for oncologists and clinical staff are essential to ensure seamless AI adoption and optimal utilization in patient care.

Abbreviations: AI, artificial intelligence; GC, gastric cancer.

### 5.1. Barriers to Artificial Intelligence Implementation

Implementing AI in GC treatment faces several challenges, notably data-related issues and the interpretability of AI models. The AI models require extensive, high-quality datasets to make accurate predictions. In GC research, data scarcity can hinder model training and validation. Additionally, biases in datasets — stemming from non-representative patient populations or inconsistent data collection methods — can lead to models that perform well in controlled settings but poorly in real-world clinical environments. Addressing these issues necessitates standardized data collection and efforts to compile diverse, comprehensive datasets (91). To overcome data limitations and bias in AI-driven GC research, several strategies have been adopted (92). Transfer learning allows models trained on large datasets from other cancers to be adapted for GC, reducing the need for extensive labeled data. Federated learning (FL) enables institutions to collaboratively train models without sharing patient data, improving generalizability and privacy. Additionally, synthetic data generation using generative adversarial networks (GANs) and variational autoencoders (VAEs) augments datasets by producing realistic samples, helping to balance classes and improve model robustness. These approaches collectively enhance the accuracy and clinical relevance of AI models in GC (92-94). Additionally, many AI models, particularly those based on DL, operate as "black boxes," providing predictions without transparent reasoning. This lack of interpretability poses a significant barrier in clinical settings, where understanding the rationale behind a recommendation is crucial for physician trust and patient safety (95, 96). Developing interpretable AI models or incorporating explainability features is essential to facilitate their integration into GC treatment protocols. Overcoming these challenges is vital for the successful application of AI in GC therapy, ensuring that technological advancements translate into tangible clinical benefits.

A key challenge in applying AI to GC research is the "black box" nature of complex models, which often lack interpretability. This makes it difficult for clinicians to trust AI-generated predictions. Explainable AI (XAI) techniques help address this by improving transparency and enabling a clearer understanding of model decisions, supporting more informed clinical use (95, 97). Various XAI methods have been developed to improve the transparency of AI models in oncology. SHAP (Shapley Additive Explanations) clarifies how input features like biomarkers influence predictions, while LIME (Local Interpretable Model-agnostic Explanations) simplifies model behavior by testing how small input changes affect outcomes. Attention mechanisms in deep learning (DL) also help by pinpointing important areas in medical images or genomic data, enhancing clinical interpretability (98, 99). Incorporating explainable AI into GC research enhances model transparency, helping oncologists better trust and apply these tools in practice. By making predictions more interpretable, XAI bridges the gap between complex algorithms and clinical decision-making. Continued focus on explainability will ensure AI delivers both accurate and meaningful insights for personalized cancer care.

### 5.2. Bridging Research and Clinical Practice

Addressing these challenges necessitates interdisciplinary collaboration between AI researchers and oncologists. Such partnerships can ensure that AI models are developed with clinical relevance and are tailored to the specific needs of GC treatment. For example, a collaboration between the Institute of Cancer Research and clinical experts led to the development of an AI test capable of predicting effective cancer drug combinations, demonstrating the potential of interdisciplinary efforts (100). Furthermore, integrating AI into clinical workflows requires joint efforts to validate AI tools, establish guidelines, and provide training for healthcare providers. A consensus statement by the American Society for Gastrointestinal Endoscopy highlighted the importance of such collaborative efforts in understanding and implementing AI in clinical practice (101).

In conclusion, while AI offers promising avenues for advancing GC treatment, overcoming implementation barriers and fostering interdisciplinary collaboration are crucial steps toward realizing its full potential in clinical settings. Despite its promise in GC treatment, AI faces several barriers to clinical adoption. Many clinicians remain cautious due to limited model transparency and lack of validation across diverse patient groups (102). Explainable AI methods like SHAP and LIME can help build trust by clarifying predictions (103). Regulatory compliance also poses a challenge, as AI tools must meet strict Food and Drug Administration (FDA) and European Medicines Agency (EMA) guidelines (104). Standardized validation protocols are essential for broader clinical acceptance (105). Implementation costs — covering infrastructure, training, and data management — are another hurdle (106), but cost-effectiveness analyses may support long-term adoption. Finally, ongoing education and training for clinical teams are key to effective AI integration (107). Addressing these challenges will improve AI uptake and patient outcomes in GC care.

### 5.3. Future Potential of Artificial Intelligence in Precision Oncology

Integrating AI with precision oncology offers new insights into drug resistance in GC. Advances in single-cell and spatial transcriptomics reveal tumor heterogeneity and cell interactions. The AI models using these data can better predict treatment responses and resistance, as shown in studies analyzing GC with peritoneal metastasis (108). Spatial transcriptomics has enabled mapping of the tumor microenvironment in GC, offering spatial context to gene expression. When combined with AI, it helps identify novel biomarkers and therapeutic targets, improving treatment precision (109). Developing effective AI models requires diverse datasets, but privacy concerns often hinder data sharing. Federated learning (FL) addresses this by allowing model training across institutions without centralizing data. In GC, it has been used to identify high-risk patients for recurrence, showing promise in improving predictive accuracy (94). Additionally, integrating generative AI with FL has shown promise in privacy-preserved sequence-based stomach adenocarcinoma detection, further underscoring the applicability of FL in GC research (110).

In conclusion, integrating spatial and single-cell transcriptomics with AI and FL represents a cutting-edge approach in GC precision oncology. These advances offer deeper insights into drug resistance and support the development of personalized therapies to enhance patient outcomes.

### 5.4. Clinical Validation of Artificial Intelligence in Gastric Cancer

Although AI holds great potential for identifying drug resistance in GC, clinical validation is essential for real-world use. Several studies have shown that AI models can effectively predict therapy response and guide treatment decisions. However, integrating these tools into routine care remains challenging. Clinical case studies are key to confirming their reliability in personalized treatment. For example, Zhang et al. developed a deep learning model using CT images to predict resistance to neoadjuvant chemotherapy in locally advanced GC, achieving AUCs of 0.808, 0.755, and 0.752 in validation cohorts (111). Metabolomic profiling has been instrumental in exploring chemotherapy resistance in GC. Studies have identified metabolic shifts in GC cells exposed to 5-FU and trastuzumab, revealing potential biomarkers of resistance (112, 113). While not AI-driven, these findings support future development of AI models using metabolomic data to predict treatment outcomes. Additionally, a machine learning study identified a 10-metabolite signature for GC diagnosis and risk stratification, offering insights into potential resistance to standard chemotherapy (114). Advances in AI are enhancing the analysis of the TME, offering insights into drug resistance and outcomes in GC. Chen et al. developed an AI method linking TME features to prognosis (115). Similarly, the PLATFORM trial (NCT02678182) used AI with multiplex immunofluorescence images to distinguish responders from non-responders to chemotherapy and immune checkpoint inhibitors, highlighting markers like FOXP3+ and CD8+PD1+ T cells (79). These findings show AI’s potential to personalize therapy and guide clinical decisions in GC.

### 5.5. Ethical and Legal Challenges in Artificial Intelligence-Driven Gastric Cancer Treatment

While AI offers promising tools for managing drug resistance in GC, its clinical use raises important ethical and legal concerns. A key issue is bias in models trained on incomplete or non-representative data, which can lead to unfair treatment recommendations and worsen healthcare disparities. Ensuring fairness requires diverse, high-quality datasets and the use of bias-detection methods during model development (116). Patient privacy and data security are essential in AI-driven GC treatment. Although AI relies on large patient datasets, handling this sensitive information poses risks of misuse. Compliance with regulations like HIPAA and GDPR is critical for protecting confidentiality. Federated learning offers a solution by enabling model training across institutions without sharing raw data, thus minimizing privacy concerns (117). Legal liability is a key concern in AI-driven healthcare. When an AI tool gives a wrong treatment recommendation, it's unclear whether the physician, institution, or developer is accountable. Unlike traditional tools, AI systems evolve with new data, complicating oversight. Clear accountability and legal frameworks are essential for safe clinical use (118). The AI regulatory pathways differ across regions, leading to a fragmented landscape. Although agencies such as the FDA and EMA are developing guidelines, standardization remains limited. The absence of unified validation protocols complicates approval and delays clinical use. Establishing global frameworks is essential for integrating AI into oncology (105). Addressing these ethical and legal challenges will enable safe, fair, and accountable use of AI in GC treatment, ensuring patient privacy and equity in clinical decisions.

## 6. Future Directions

The AI is revolutionizing precision therapy in GC by enhancing therapeutic stratification, optimizing drug combinations, designing adaptive therapies, and integrating with clinical workflows.

### 6.1. Next-Generation Artificial Intelligence Models

Integrating explainable AI (XAI) into GC research helps overcome the "black box" issue, increasing clinician trust. By clarifying AI decisions, XAI supports better understanding and validation. A study on gastrointestinal cancers shows its value in improving diagnosis and treatment planning (119). Artificial intelligence models are being developed to predict how drug resistance evolves in GC by analyzing tumor genetics and cell responses. Tools like PERCEPTION use single-cell transcriptomics to anticipate treatment outcomes and guide personalized therapy (8). Next-generation AI models offer promise for improving GC treatment by predicting resistance patterns and enabling tailored interventions.

### 6.2. Integrating Multi-Omics with Real-Time Patient Data

Combining static genomic insights with dynamic clinical data through AI enables a comprehensive understanding of drug resistance mechanisms in GC. Integrative models that analyze genomic, transcriptomic, and proteomic data alongside real-time patient information can identify biomarkers predictive of resistance and monitor treatment responses. Such approaches facilitate the development of adaptive therapies tailored to the evolving molecular landscape of individual patients' tumors.

### 6.3. Advancing Drug Discovery with Artificial Intelligence

Advancements in AI are significantly enhancing drug discovery efforts, particularly in identifying novel compounds that target resistance pathways in GC. By modeling complex biological interactions, AI can design drugs that specifically inhibit mechanisms underlying therapeutic resistance. For instance, AI-driven platforms have been developed to design multi-target drugs, which can simultaneously disrupt multiple pathways involved in cancer progression and resistance. Additionally, AI models have been employed to discover potential anti-cancer drug targets through the analysis of synthetic lethality, offering new avenues for treatment development. These AI-driven approaches hold promise for accelerating the development of effective therapies against drug-resistant GC (120, 121).

## 7. Conclusions

Artificial intelligence is revolutionizing GC treatment by providing innovative approaches to address drug resistance. It integrates multi-omics data to identify biomarkers, analyze disrupted pathways, and predict resistance mechanisms. Machine learning (ML) models reveal how GC cells adapt to therapies, while AI-driven drug discovery and real-time adaptive therapy systems enhance treatment precision. Future advancements in explainable AI, federated learning, and single-cell transcriptomics promise to bridge research and clinical practice. By overcoming challenges like data quality and model interpretability, AI is set to deliver personalized, adaptive treatments, improving outcomes and quality of life for GC patients.

## Data Availability

The dataset presented in the study is available on request from the corresponding author during submission or after publication.
